# The Challenges of Genome-Wide Interaction Studies: Lessons to Learn from the Analysis of HDL Blood Levels

**DOI:** 10.1371/journal.pone.0109290

**Published:** 2014-10-20

**Authors:** Elisabeth M. van Leeuwen, Françoise A. S. Smouter, Tony Kam-Thong, Nazanin Karbalai, Albert V. Smith, Tamara B. Harris, Lenore J. Launer, Colleen M. Sitlani, Guo Li, Jennifer A. Brody, Joshua C. Bis, Charles C. White, Alok Jaiswal, Ben A. Oostra, Albert Hofman, Fernando Rivadeneira, Andre G. Uitterlinden, Eric Boerwinkle, Christie M. Ballantyne, Vilmundur Gudnason, Bruce M. Psaty, L. Adrienne Cupples, Marjo-Riitta Järvelin, Samuli Ripatti, Aaron Isaacs, Bertram Müller-Myhsok, Lennart C. Karssen, Cornelia M. van Duijn

**Affiliations:** 1 Genetic Epidemiology Unit, Department of Epidemiology, Erasmus Medical Center, Rotterdam, the Netherlands; 2 Max Planck-Institute of Psychiatry, Munich, Germany; 3 Icelandic Heart Association, Kopavogur, Iceland and University of Iceland, Reykjavik, Iceland; 4 Laboratory of Epidemiology, Demography, and Biometry, National Institute on Aging, National Institutes of Health, Bethesda, Maryland, United States of America; 5 Cardiovascular Health Research Unit and Department of Medicine, University of Washington, Seattle, WA, United States of America; 6 Department of Biostatistics, Boston University School of Public Health, Boston, Massachusetts, United States of America; 7 Institute for Molecular Medicine Finland (FIMM), University of Helsinki, Helsinki, Finland; 8 Department of Clinical Genetics, Erasmus Medical Center, Rotterdam, the Netherlands; 9 Department of Epidemiology, Erasmus Medical Center, Rotterdam, the Netherlands; 10 Departments of Epidemiology and Internal Medicine, Erasmus Medical Center, Rotterdam, the Netherlands; 11 University of Texas Health Science Center School of Public Health, Human Genetics Center, Houston, TX, United States of America; 12 Department of Medicine, Baylor College of Medicine, Houston, TX, United States of America; 13 Departments of Epidemiology, Medicine and Health Services, University of Washington, Seattle, WA, United States of America and Group Health Research Institute, Group Health, Seattle, WA, United States of America; 14 Framingham Heart Study, Framingham, Massachusetts, United States of America; 15 Department of Epidemiology and Biostatistics, MRC Health Protection Agency (HPA) Centre for Environment and Health, School of Public Health, Imperial College London, London, United Kingdom; 16 National Institute for Health and Welfare, Oulu, Finland; 17 Biocenter Oulu, University of Oulu, Oulu, Finland; 18 Institute of Health Sciences, University of Oulu, Oulu, Finland; 19 Unit of Primary Care, Oulu University Hospital, Oulu, Finland; 20 Wellcome Trust Sanger Institute, Hinxton, Cambridge, United Kingdom; 21 Hjelt Institute, University of Helsinki, Helsinki, Finland; 22 Munich Cluster für Systems Neurology (Synergy), Munich, Germany; 23 Institute of Translational Medicine, University of Liverpool, Liverpool, United Kingdom; Johns Hopkins University, United States of America

## Abstract

Genome-wide association studies (GWAS) have revealed 74 single nucleotide polymorphisms (SNPs) associated with high-density lipoprotein cholesterol (HDL) blood levels. This study is, to our knowledge, the first genome-wide interaction study (GWIS) to identify SNP×SNP interactions associated with HDL levels. We performed a GWIS in the Rotterdam Study (RS) cohort I (RS-I) using the GLIDE tool which leverages the massively parallel computing power of Graphics Processing Units (GPUs) to perform linear regression on all genome-wide pairs of SNPs. By performing a meta-analysis together with Rotterdam Study cohorts II and III (RS-II and RS-III), we were able to filter 181 interaction terms with a *p*-value<1 · 10^−8^ that replicated in the two independent cohorts. We were not able to replicate any of these interaction term in the AGES, ARIC, CHS, ERF, FHS and NFBC-66 cohorts (*N*_total_ = 30,011) when adjusting for multiple testing. Our GWIS resulted in the consistent finding of a possible interaction between rs774801 in *ARMC8* (ENSG00000114098) and rs12442098 in *SPATA8* (ENSG00000185594) being associated with HDL levels. However, *p-*values do not reach the preset Bonferroni correction of the *p*-values. Our study suggest that even for highly genetically determined traits such as HDL the sample sizes needed to detect SNP×SNP interactions are large and the 2-step filtering approaches do not yield a solution. Here we present our analysis plan and our reservations concerning GWIS.

## Introduction

To date, genome-wide association studies (GWAS) have revealed 95 genetic loci associated with lipid levels in human plasma. Of these, 74 SNPs were associated with high-density lipoprotein cholesterol (HDL) levels [1]–[5]. Together, these 47 SNPs explain approximately 25% of the heritability of HDL levels. Although the largest meta-analysis of plasma lipid concentrations [4] to date, already included more than 100,000 individuals of European descent, it is expected that with increasing sample size and larger, better reference panels for imputation, more variants will be found to be associated with HDL levels, probably resulting in an increase of the explained heritability. Nevertheless, single SNP effects may not fully explain the heritability of HDL levels. Genetic processes like DNA methylation, histone modification and interactions between SNPs are also potential candidates determining HDL levels [6]–[9]. A previous large study did not find evidence of gene-environment interactions influencing HDL levels, although this might also play a role with other environmental factors [10]. We defined interactions between SNPs as a departure from a linear statistical model allowing for the additive marginal effects of both SNPs. Persistent evidence for interacting loci involved in lipid metabolism comes from experimental animal research in which various loci interact with each other [11].

Based on the loci for HDL levels identified to date, finding evidence for SNP×SNP interactions in humans has proven to be difficult. Ma *et al.* [8] identified a significant association interaction between a locus within the *HMGCR* gene (ENSG00000113161) and a locus near the *LIPC* gene (ENSG00000166035) in relation to HDL cholesterol. Furthermore, Turner *et al.* [9] found 8 SNP×SNP interactions to be associated with HDL levels of which the strongest model included an interaction between *LPL* (ENSG00000175445) and *ABCA1* (ENSG00000165029). These studies suggest that SNP×SNP interactions can indeed also explain some of the heritability of HDL levels in humans. However, only loci were studied that had previously been successfully replicated in GWAS of lipid levels, thus motivating a genome-wide search for interactions associated with HDL levels.

Genome-wide searches for associations between phenotypes and SNP×SNP interactions have been hampered by the computation time needed for testing all unique pairs of SNPs, given by *N*_SNPs_(*N*_SNPs_-1)/2, with *N*_SNPs_ the total number of SNPs. Consequently, the time for testing all interaction terms is proportional to *N*_SNPs_^2^, translating into months of computation time. Modern Graphics Processing Units (GPUs) are optimised for highly parallel computation tasks and are well-suited to replace regular processors (Central Processing Units or CPUs) for these kind of tasks. The GLIDE software package [12] makes use of GPUs to perform linear regression for all pairs of SNPs. In this study, we aim to identify SNP×SNP interactions for HDL levels in the Rotterdam Study cohort I (RS-I) using GLIDE. The most significant interactions terms in RS-I are first filtered by a meta-analysis in cohorts II and III of the Rotterdam Study (RS-II and RS-III, respectively). The resulting interactions were subsequently sent for replication in the CHARGE cohorts (AGES, ARIC, CHS, ERF, FHS) and the NFBC-66 cohort. We also tested whether the identified interaction terms are associated to dyslipidemia treatment within the cohorts of the Rotterdam Study.

## Results

### GWIS with GLIDE in RS-I

**Figure 1:**
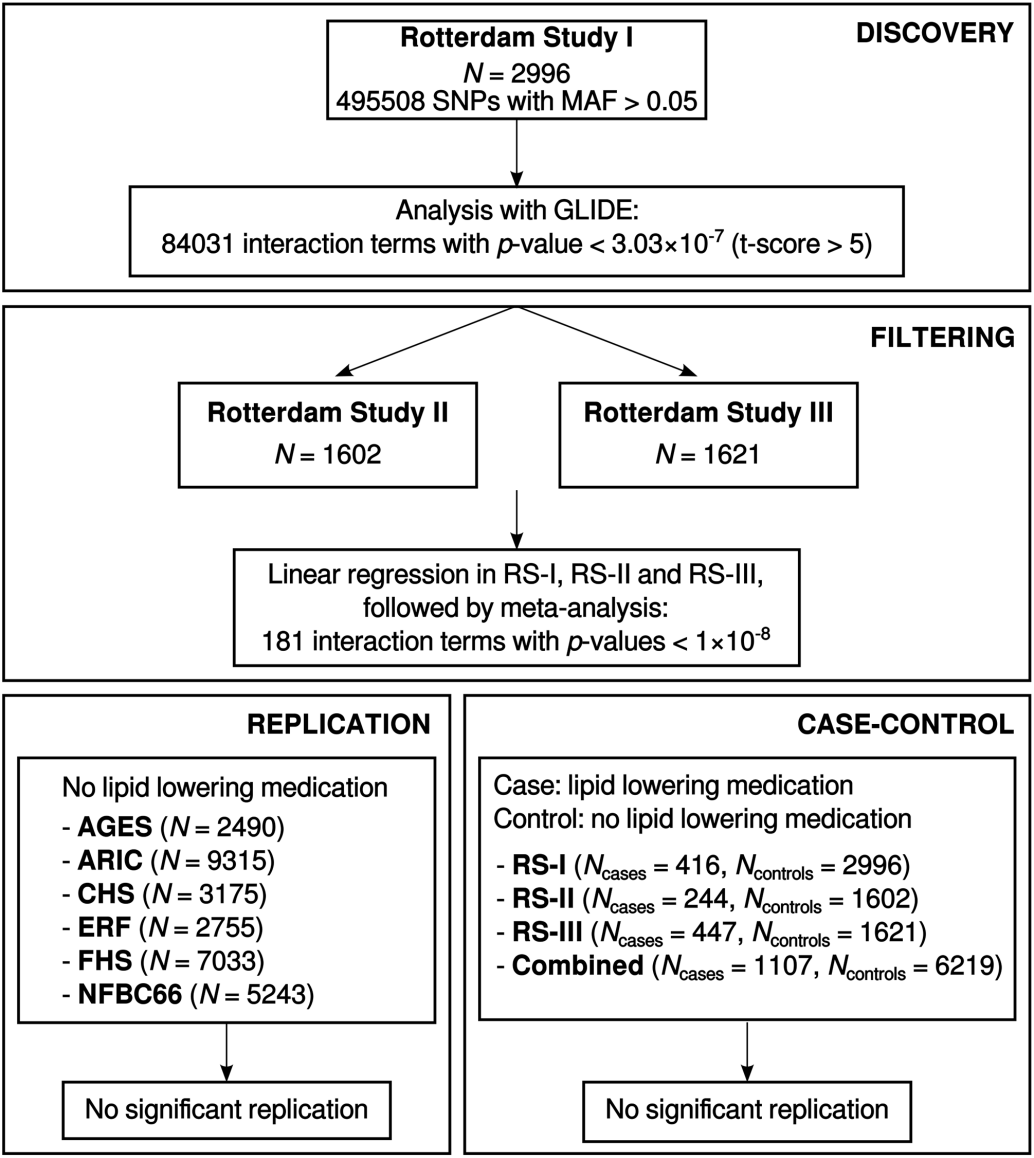
Flow diagram overview of the analysis plan.

Figure 1 shows a flow diagram illustrating the analysis plan. A total of 495,508 genotyped SNPs that passed quality control, had a Minor Allele Frequency (MAF) >0.05 in the sample of 2,996 individuals from RS-I, and were also genotyped in RS-II and RS-III were used to identify SNP×SNP interactions associated with HDL using GLIDE. For this analysis the HDL levels after adjustment for sex and age were normalised around zero as this is a requirement of GLIDE. This resulted in 84,031 SNP×SNP interactions with an absolute value of the *t*-score >5 (i.e. *p*<6.06 · 10^−7^).

### Filtering of interaction terms by a meta-analysis of RS-I, RS-II and RS-III

Using linear regression we calculated the regression coefficient β_int_ for the interaction term, the standard errors and the *p*-values for the 84,031 interaction terms in RS-I (*N* = 2,996), RS-II (*N* = 1,602) and in RS-III (*N* = 1,621). For these analyses the HDL levels after adjustment for sex and age were normalised around zero since this was done in RS-I in the initial analysis with GLIDE as this is a requirement of GLIDE. The calculated β_int_ and standard errors were used to meta-analyse the association between each of the 84,031 interaction terms and HDL levels. After meta-analysis, 181 interaction terms with a *p-*value below 1 · 10^−8^ remain, of which 5 interaction terms with a *p*-value less than 1 · 10^−10^. The pooled β_int_ for the 84,031 interaction terms range from −0.507 to 0.746. The 181 interaction terms with a *p*-value less than 1 · 10^−8^ were taken forward for replication, see Table S1. The number of unique interaction terms for replication was reduced to 132 by filtering on linkage disequilibrium (LD) between interaction terms (*R* ^2^>0.8). Consequently, the *p*-value for replication after Bonferroni correction is 3.79 · 10^−4^. We also calculated the β_int_ of RS-I, RS-II and RS-III for these 181 interaction terms using linear regression with the unscaled phenotype to compare these with the β_int_ within the replication cohorts.

### Replication of SNP×SNP interactions

**Figure 2:**
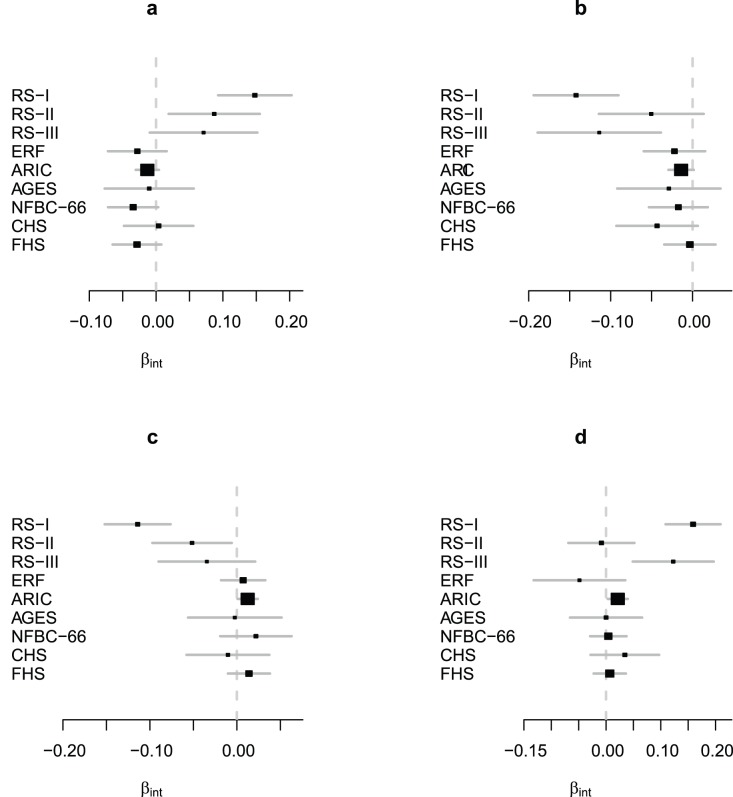
The forest plots for β_int_ of the four most significant interaction terms after meta-analysis of the replication cohorts: rs2315598-rs2853228 (a), rs6848132-rs7863451 (b), rs3756856-rs11758333 (c) and rs4596126-rs11676467 (d). Although the analysis in the discovery and the filtering was done with scaled phenotypes, for these forest plots, the HDL levels are not scaled in the Rotterdam Study cohorts.

Replication was conducted in 6 cohorts: AGES, ARIC, CHS, ERF, FHS and NFBC-66. In the replication cohorts only individuals not on lipid-lowering medication were included, with the exception of AGES, see Table 1. In AGES, ARIC, CHS, ERF and FHS, 8, 7, 7, 10 and 7 interaction terms, respectively, could not be tested for replication since one or both of the SNPs in the interaction term had not been genotyped or imputed. In NFBC-66 all interaction terms could be tested for replication. A total of 170 out of the 181 interactions could be tested for replication in all six cohorts. None of the interaction terms reached a significant *p*-value after Bonferroni correction (3.79 · 10^−4^) in any of the replication cohorts and after meta-analysis of all replication cohorts. Four interaction terms reached nominal significance at *p* = 0.05, see Figure 2. The lowest *p*-value for β_int_ after meta-analysis of all replication cohorts (*N* = 30,011) was 7.57 · 10^−3^ for the interaction between rs2315598 (chromosome 2, position 132,994,224, gene *GPR39* (ENSG00000183840)) and rs2853228 (chromosome 8, position 103,296,258, gene *RRM2B* (ENSG00000048392)). The second lowest *p*-value for β_int_ after meta-analysis of all replication cohorts (*N* = 30,011) was 8.1 · 10^−3^ for the interaction between rs6848132 (chromosome 4, position 93,460,610, gene *GRID2* (ENSG00000152208)) and rs7863451 (chromosome 9, position 129,112,065, gene *GARNL3* (ENSG00000136895)). The β_int_ is negative in all nine cohorts. Table 2 shows the 20 interaction terms with the lowest *p*-values. Five of these terms are interactions between an intergenic locus at chromosome 6, situated between the *TCP11* (ENSG00000124678) and *SCUBE3* (ENSG00000146197) genes, and a locus at the same chromosome in the *SOBP* gene (ENSG00000112320) which are in LD with each other (*R* ^2^>0.872).

**Table 1:** Baseline characteristics for discovery and replication cohorts.

	Study	Country of origin	N (% male)
RS-I	Rotterdam Study cohort I	Netherlands	2996 (57.7)
RS-II	Rotterdam Study cohort II	Netherlands	1602 (54.9)
RS-III	Rotterdam Study cohort III	Netherlands	1621 (58.3)
AGES	Age, Gene/Environment Susceptibility Study	Iceland	3219 (42.0)
ARIC	Atherosclerosis Risk in Communities Study	United States	9315 (46.9)
CHS	Cardiovascular Health Study	Americans of European descent	3175 (40)
ERF	Erasmus Rucphen Family study	Netherlands	2755 (44.7)
FHS	Framingham Heart Study	Americans of European descent	7033 (46)
NFBC-66	Northern Finland Brith Cohort 1966	Finland	5243 (47.98)
	**Mean age (SD), years**	**HDL cholesterol (SD), mmol/L**	**lipid lowering medication users**
RS-I	66.2 (7.2)	1.39 (0.39)	excluded
RS-II	64.7 (8.1)	1.38 (0.37)	excluded
RS-III	55.6 (5.7)	1.47 (0.44)	excluded
AGES	76.4 (5.5)	1.58 (0.45)	included (22.6%)
ARIC	54.3 (5.7)	1.31 (0.43)	excluded
CHS	72.5 (5.4)	1.43 (0.41)	excluded
ERF	48.9 (14.4)	1.27 (0.36)	excluded
FHS	37.5 (9.6)	1.37 (0.40)	excluded
NFBC-66	31 (0)	1.56 (0.38)	excluded

**Table 2:** The top 20 interaction terms after replication.

Interaction	Direction of β_int_ **									Meta-analysis of RS-I, RS-II and RS-III			Meta-analysis of replication cohorts		
										β_int_	SE	*p*-value	β_int_	SE	*p*-value
rs2315598–rs2853228	+	+	+	−	−	−	−	+	−	0,1123	0,0193	5,91E-9	−0,0178	0,0067	0,0076
rs6848132–rs7863451	−	−	−	−	−	−	−	−	−	−0,1077	0,0181	2,76E-9	−0,0158	0,0060	0,0081
rs3756856–rs11758333*	−	−	−	+	+	−	+	−	+	−0,0769	0,0132	5,33E-9	0,0112	0,0047	0,0160
rs4596126–rs11676467	+	−	+	−	+	+	+	+	+	0,0973	0,0175	2,47E-8	0,0137	0,0068	0,0436
rs871849–rs9672547	−	−	−	−	−	−	−	−	+	−0,0820	0,0141	5,86E-9	−0,0092	0,0048	0,0557
rs5018943–rs7023148	−	−	−	+	−	−	−	−	−	−0,0944	0,0159	2,96E-9	−0,0132	0,0069	0,0558
rs2280518–rs9875407	+	+	+	+	+	+	+	+	+	0,1393	0,0242	9,22E-9	0,0161	0,0085	0,0574
rs754950–rs10926977	+	+	+	−	−	−	+	−	−	0,0876	0,0148	2,92E-9	−0,0103	0,0055	0,0608
rs2301818–rs7537266	−	−	−	−	+	−	+	+	+	−0,0905	0,0156	6,43E-9	0,0098	0,0053	0,0626
rs10816852–rs10911901	+	+	+	+	+	+	+	−	−	0,1491	0,0257	6,45E-9	0,0135	0,0075	0,0734
rs645925–rs12231356	+	+	+	−	−	−	−	+	+	0,1102	0,0184	2,04E-9	−0,0112	0,0063	0,0749
rs820065–rs7762721*	−	−	−	−	−	−	+	−	+	−0,0768	0,0132	5,36E-9	−0,0082	0,0046	0,0782
rs7214582–rs17075071	+	+	+	−	−	+	−	−	−	0,2005	0,0349	9,40E-9	−0,0190	0,0109	0,0817
rs774801–rs12442098	−	−	−	−	−	+	−	+	−	−0,0960	0,0167	8,57E-9	−0,0101	0,0059	0,0866
rs693–rs4677039	+	+	+	+	+	+	+	+	−	0,0570	0,0102	2,07E-8	0,0057	0,0034	0,0928
rs3756856–rs7762721*	−	−	−	−	+	+	−	−	+	−0,0836	0,0131	1,82E-10	0,0078	0,0046	0,0928
rs2242312–rs11190870	+	+	+	+	+	−	+	−	−	0,1238	0,0205	1,65E-9	0,0112	0,0067	0,0951
rs3756856–rs6940398*	−	−	−	−	+	−	−	+	+	−0,0793	0,0131	1,61E-9	0,0078	0,0047	0,0982
rs2316640–rs10973877	+	+	+	+	−	+	−	−	+	0,0729	0,0122	2,08E-9	−0,0064	0,0039	0,0990
rs2234044–rs7762721*	−	−	−	+	+	−	−	−	+	−0,0807	0,0132	1,10E-9	0,0075	0,0046	0,1044

The interactions are sorted based on the *p*-value of the interaction term β_int_. The HDL levels are adjusted for sex and age. The phenotypes of the Rotterdam Study are not scaled around zero.

*Interaction terms in LD with each other (*R* ^2^>0.872).

**order of the directions: AGES, ARIC, CHS, ERF, FHS, NFBC-66, RS-I, RS-II, RS-III. The meta-analysis was done with fixed effects.

Individuals with high levels of low-density lipoprotein (LDL) or low levels of HDL are treated with lipid-lowering medication. The 181 selected interaction terms were also tested to see whether their presence might explain the use of lipid-lowering medication and therefore the extreme lipid levels. To this end the individuals of the Rotterdam Study in the discovery and filtering stage were used as controls, and the individuals of the Rotterdam Study who use lipid-lowering medication were used as cases. Table 3 shows the 20 interaction terms with the lowest *p*-values for β_int_ after testing in the three cohorts of the Rotterdam Study combined. The interaction between rs6442460 (chromosome 3, position 14,551,071, gene *GRIP2* (ENSG00000144596)) and rs10914332 (chromosome 1, position 31,471,589, gene *NKAIN1* (ENSG00000084628)) had the lowest *p*-value (*p* = 3.98 · 10^−3^).

**Table 3:** The top 20 interaction terms after case-control studies in RS-I, RS-II and RS-III separate and combined.

Interaction	Case-control in RS-I			Case-control in RS-II			Case-control in RS-III			Case-control RS combined		
	β_int_	SE	*p*-value	β_int_	SE	*p*-value	β_int_	SE	*p*-value	β_int_	SE	*p*-value
rs6442460–rs10914332	−0,5227	0,286	0,0673	−0,4306	0,396	0,2775	−0,6454	0,305	0,0346	−0,53	0,1841	0,00398
rs2146043–rs11124513	−0,25056	0,172	0,1452	−0,2557	0,249	0,3039	−0,356	0,179	0,0472	−0,302	0,1097	0,00591
rs774801–rs12442098 *1	0,35367	0,18	0,0497	0,4151	0,245	0,0898	0,1702	0,21	0,4188	0,293	0,1175	0,01267
rs3006496–rs17729021	0,25625	0,135	0,0579	0,198	0,186	0,2872	0,1344	0,147	0,359	0,208	0,087	0,01694
rs754970–rs2306478 *2	−0,17851	0,153	0,2422	−0,17	0,181	0,3469	−0,2445	0,137	0,0741	−0,202	0,0871	0,0205
rs754970–rs3775972 *2	−0,17089	0,152	0,2614	−0,1678	0,18	0,3501	−0,2482	0,137	0,0695	−0,199	0,0869	0,02226
rs5770418–rs17234336	−0,94012	0,422	0,0258	−0,6081	0,53	0,2513	−0,2122	0,318	0,5047	−0,489	0,2213	0,02703
rs7631734–rs12442098 *1	0,31044	0,181	0,0855	0,4119	0,247	0,0959	0,1156	0,211	0,5845	0,256	0,1179	0,03002
rs2242312–rs11190870	0,24296	0,226	0,2833	0,4927	0,323	0,1275	0,2608	0,231	0,2594	0,304	0,1437	0,03462
rs4861849–rs17123865	−0,04394	0,415	0,9156	0,8862	0,499	0,0757	0,7524	0,338	0,0262	0,446	0,2265	0,04902
rs754950–rs4658547 *3	−0,15255	0,162	0,3451	−0,2093	0,232	0,367	−0,2082	0,174	0,2314	−0,202	0,1045	0,05368
rs1203791–rs10496556	−0,29897	0,151	0,0479	−0,0571	0,189	0,7629	−0,1164	0,155	0,4514	−0,162	0,0923	0,07865
rs754950–rs10926977 *3	−0,15562	0,159	0,3275	−0,1923	0,229	0,4002	−0,1322	0,17	0,4372	−0,172	0,1026	0,09409
rs900654–rs10195135 *4	0,07114	0,161	0,6594	0,3194	0,209	0,1267	0,2456	0,17	0,1477	0,168	0,1006	0,09566
rs10195135–rs10872670 *4	0,1016	0,16	0,5246	0,2728	0,21	0,1947	0,2195	0,168	0,192	0,166	0,1003	0,09804
rs2919732–rs12759209	−0,18926	0,171	0,2683	−0,3477	0,217	0,1086	−0,0725	0,164	0,6583	−0,168	0,1022	0,10006
rs806454–rs17578868	0,22532	0,17	0,1861	0,3827	0,26	0,1404	0,093	0,177	0,5992	0,18	0,1102	0,10155
rs2013041–rs10511302	0,00345	0,143	0,9808	−0,1725	0,19	0,363	−0,305	0,157	0,0518	−0,148	0,0911	0,105
rs10494757–rs11520658	−1,77249	1,013	0,08	0,2238	0,687	0,7447	−0,536	0,564	0,3416	−0,608	0,3834	0,11292
rs1426588–rs10248926	0,1073	0,141	0,4472	0,0694	0,195	0,7214	0,2176	0,145	0,1332	0,133	0,0889	0,13558
rs2013041–rs10511302	0,00345	0,143	0,9808	−0,1725	0,19	0,363	−0,305	0,157	0,0518	−0,148	0,0911	0,105
rs10494757–rs11520658	−1,77249	1,013	0,08	0,2238	0,687	0,7447	−0,536	0,564	0,3416	−0,608	0,3834	0,11292
rs1426588–rs10248926	0,1073	0,141	0,4472	0,0694	0,195	0,7214	0,2176	0,145	0,1332	0,133	0,0889	0,13558

The interactions are sorted based on the *p*-value of the β_int_ after the case-control study in the combined data set of RS-I, RS-II and RS-III. The HDL levels are adjusted for sex and age, the residuals are not scaled around zero. Number of cases: 416 (RS-I), 244 (RS-II) and 447 (RS-III). Number of controls: 2996 (RS-I), 1602 (RS-II) and 1621 (RS-III). *1–*4 mark the interaction terms that are in high LD with each other: *R* ^2^>0.913.

Three interaction terms overlap between the top 20 hits after the replication and the top 20 hits after the case-control test, as shown in Table 4. None of the SNPs of these interaction terms are in high LD with each other (*R* ^2^>0.8). The interaction between rs754950 and rs10926977 has an opposite effect direction after the meta-analysis in the Rotterdam Study cohorts compared to the one after meta-analysis in the replication cohorts and thus will probably be a false-positive finding. The second interaction term (between rs2242312 and rs11190870) had a positive effect on HDL, but increases the risk of lipid lowering medication which is counter-intuitive and consequently this interaction term is likely a false-positive finding as well. The third interaction term, however, between rs774801 (chromosome 3, position 139,413,035, gene *ARMC8* (ENSG00000114098)) and rs12442098 (chromosome 15, position 95,385,874, close to gene *SPATA8* (ENSG00000185594)) has a negative effect on HDL combined with a positive effect on the use of lipid lowering medication. Although this last interaction term is not replicated, the directions of the effects are consistent since this interaction lowers the HDL level and increases the chance of using lipid lowering medication.

**Table 4:** The overlap between the top 20 interaction terms after replication and case-control analysis.

Interaction	Meta-analysis of RS-I, RS-II and RS-III			Meta-analysis of replication cohorts			Case-control in combined RS		
	β_int_	SE	*p*-value	β_int_	SE	*p*-value	β_int_	SE	*p*-value
rs754950–rs10926977	0,0876	0,0148	2,92E-009	−0,01028	0,00548	0,06078	−0,172	0,1026	0,09409
rs2242312–rs11190870	0,1238	0,0205	1,65E-009	0,01121	0,00671	0,09511	0,304	0,1437	0,03462
rs774801–rs12442098	−0,096	0,0167	8,57E-009	−0,01009	0,00588	0,08656	0,293	0,1175	0,01267

### Power calculations

**Figure 3:**
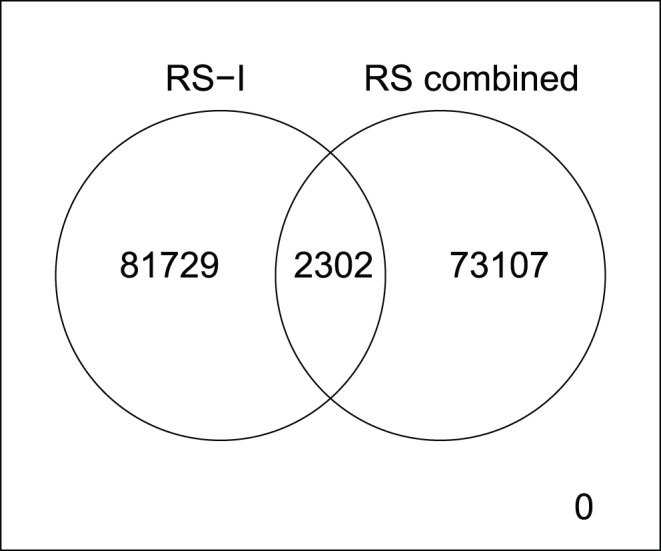
The smallest detectable effect with the current sample size of 2,996 individuals at 80% (a), 50% (b) and 1% (c) power levels.

As none of the findings replicated, we explored the statistical power of our analyses. Figure 3 shows the power calculations using the program G*Power [21], [22]. With our current sample size of 2,996 individuals the smallest detectable effect will be 0.11, 0.095 and 0.05 when the type I error is less than 1 · 10^−7^ and the type 2 error is 20% (power is 80%), 50% (power is 50%) and 99% (power is 1%), respectively.

## Discussion

Here we presented the, to our knowledge, first GWIS of HDL levels in blood. Our study shows that in a single population a GWIS results in 84,031 SNP×SNP interactions associated with HDL levels (*p*-value<6.06 · 10^−7^). Our two-step approach to filter these SNP×SNP interactions using two additional cohorts resulted in 181 interactions with a *p*-value below 1 · 10^−8^. Although some reached nominal significance, none of these interactions terms were significantly replicated in a meta-analysis of 30,011 samples when adjusting for multiple testing. We also did not find a significant association between any of the interaction terms and treatment with lipid lowering medication in the cohorts of the Rotterdam Study after adjustment for multiple testing.

To our knowledge, no other GWIS studies with HDL exist with which we can compare our results. However, we did try to replicate previously published SNP×SNP interactions. We adjusted for the same covariates as the authors did, except for smoking, which was used as a covariate by Turner *et al.* [9]. Turner *et al.* published an interaction between rs253 and rs2515614 associated with HDL, however, the *p*-values of β_int_ after testing this interaction term were 0.986, 0.189 and 0.594 in the RS-I, RS-II and RS-III cohorts, respectively. The *p*-value of β_int_ after meta-analysing this interaction term is 0.614. The interaction term between rs3846662 and rs1532085, as published by Ma *et al.* [8], only replicated in RS-III (*p* = 0.0214), but not in RS-I (*p* = 0.212) or RS-II (*p* = 0.162). The *p*-value of β_int_ after meta-analysing this interaction term is 0.335.

**Figure 4:**
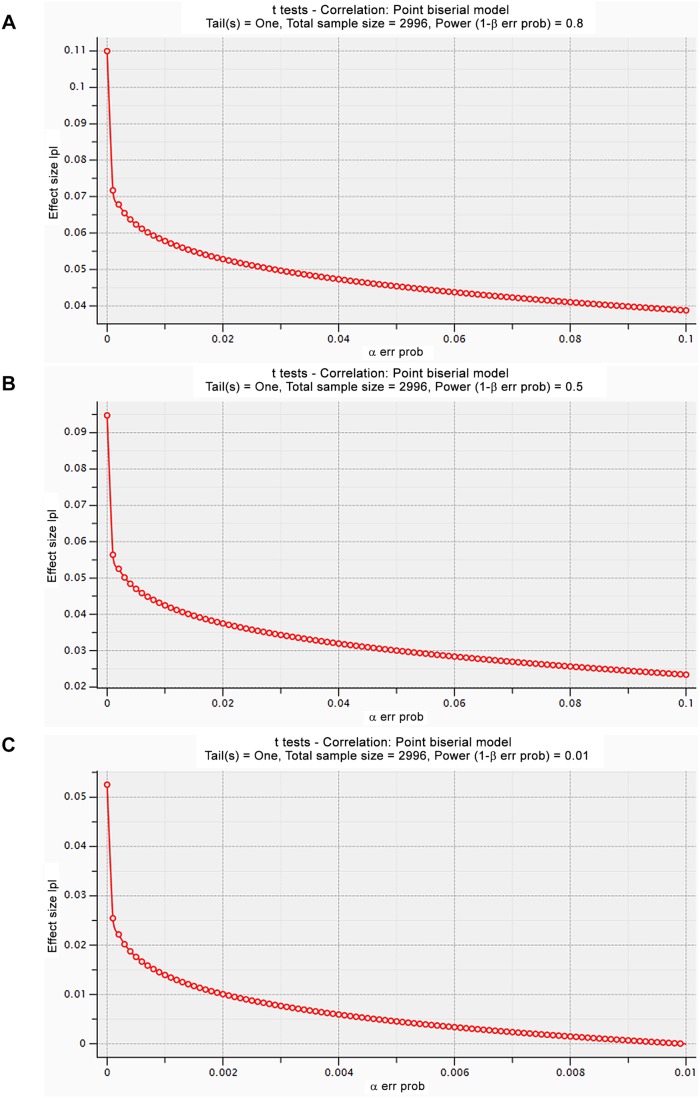
The overlap between the interaction terms with *p*-value<3.03 · 10^−7^ after a GWIS with GLIDE in RS-I only and after a GWIS with GLIDE in RS-I, RS-II and RS-III combined.

There can be multiple reasons why we were not able to uncover SNP×SNP interactions using a hypothesis-free approach. First, in this study we selected only common variants (MAF>0.05) which were genotyped in the Rotterdam Study. We chose these variants to avoid false positive findings in rare variants. Furthermore, the power to detect interaction terms with rare variants is low since our sample size in the two-stage discovery phase was 6,219. A second limitation that we chose to only investigate genotyped SNPs instead of imputed SNPs. Therefore, we may have missed true positive causal SNPs which are not on the genotyping array. However, even with only genotyped SNPs the number of potentially true positive findings is enormous, resulting in 84,031 suggestive hits at *p* = 6.06 · 10^−7^. This prompted us to use a two-stage discovery phase in which we used the RS-II and RS-III cohorts to filter out the false positives, reducing the number of findings from 84,031 to 181. The total number of individuals in this two-step discovery phase is 6,219. This might be considered low for the identification of SNP×SNP interactions. As a commonly used rule-of-thumb, the sample size within a GWIS should be 3 to 4 times the size of GWAS. As the first GWAS identifying loci associated with HDL levels [1] included 2,758 individuals, our study is expected to be underpowered by that rule. To improve power, an alternative approach could have been to combine the three cohorts of the Rotterdam Study into an one-step discovery with GLIDE. This, however, still yielded 75,409 interactions with a *p*-value below our threshold of 6.06 · 10^−7^ as compared to the 84,031 interactions seen in the RS-I only GWIS, see Figure 4. It should be noted that both numbers are well in keeping with expectations.

The proposed genome-wide significance level for GWIS is 1 · 10^−13^ [13], however, in this study we used all interaction terms with a *p*-value less than 1 · 10^−8^ for replication. We chose a much less stringent *p*-value to prevent us from missing true positives due to the relatively small sample size. However, none of the 84,031 interaction terms had a *p*-value below 1 · 10^−13^ in the separate Rotterdam Study cohorts and after meta-analysis of the three Rotterdam Study cohorts.

The success of GWAS has been its hypothesis-free approach and this worked well for studying lipids even in studies we consider small by today’s standards (1000–3000 individuals). A GWIS is now technically feasible but needs larger sample sizes. Our study shows that the number of hits is overwhelming at a *p*-value of 1 · 10^−8^. The filtering approach in a similar population did not resolve this problem. Our GWIS resulted in the consistent finding of a possible interaction between rs774801 in *ARMC8* (ENSG00000114098) and rs12442098 in *SPATA8* (ENSG00000185594) being associated with HDL levels, both in the quantitative analysis and the case-control analysis. However, *p*-values do not reach the preset Bonferroni correction of the *p*-values. Other major issues related to the sample size and apparent lack of replication also needs to be overcome.

## Methods

### Study descriptions

#### Ethics Statement

The AGES Reykjavik Study Genome Wide Association study was approved by the National Bioethics Committee (00–063) and the Data Protection Authority. The ARIC study was approved by ‘The University of Texas Health Science Center at Houston Committee for the Protection of Human Subjects’. The CHS study was approved by the following institutional review boards: Wake Forest University, University of California (Davis), Johns Hopkins University (Bloomberg School of Public Health), University of Pittsburgh, University of Washington, University of Vermont. The ERF study was approved by the Medical Ethics Committee of the Erasmus MC. The committee is constituted according to the WMO (National act medical-scientific research in human beings). The FHS was approved by the Boston University Medical Campus Institutional Review Board. The NFBC66 was approved by the Ethical Committee of the Northern Ostrobothnia Hospital District. The Rotterdam Study has been approved by the medical ethics committee according to the Population Study Act Rotterdam Study, executed by the Ministry of Health, Welfare and Sports of the Netherlands. A written informed consent was obtained from all study participants for all cohorts.

#### Discovery cohort

*Rotterdam Study cohort I (RS-I).* The Rotterdam Study is an ongoing prospective population-based cohort study, focused on chronic disabling conditions of the elderly. The study comprises an outbred ethnically homogenous population of Dutch Caucasian origin. The rationale of the study has been described in detail elsewhere [14]. In summary, 7,983 men and women aged 55 years or older, living in Ommoord, a suburb of Rotterdam, the Netherlands, were invited to participate in the first phase. Fasting blood samples were taken during the participant’s third visit to the research center.

#### Filtering cohorts

*Rotterdam Study cohort II (RS-II).* The Rotterdam Study cohort II prospective population-based cohort study comprises 3,011 residents aged 55 years and older from the same district of Rotterdam. The rationale and study designs of this cohort is similar to that of the RS-I [14]. The baseline measurements, including the fasting HDL measurements, took place during the first visit.

*Rotterdam Study cohort III (RS-III).* The Rotterdam Study cohort III prospective population-based cohort study comprised 3,932 residents aged 45 years and older from the same district of Rotterdam. The rationale and study designs of this cohort is similar to that of the RS-I [14]. The baseline measurements, including the fasting HDL measurements, took place during the first visit.

#### Replication cohorts

*Age, Gene/Environment Susceptibility (AGES Reykjavik) Study.* The Age, Gene/Environment Susceptibility (AGES Reykjavik) Study was initiated to examine genetic susceptibility and gene/environment interaction as these contribute to phenotypes common in old age, and represents a continuation of the Reykjavik Study cohort begun in 1967. The study is approved by the Icelandic National Bioethics Committee, (VSN: 00–063) and the Data Protection Authority. The researchers are indebted to the participants for their willingness to participate in the study.

*Atherosclerosis Risk in Communities (ARIC) Study.* The Atherosclerosis Risk in Communities Study (ARIC), sponsored by the National Heart, Lung, and Blood Institute (NHLBI) is a prospective epidemiologic study conducted in four U.S. communities. ARIC is designed to investigate the causes of atherosclerosis and its clinical outcomes, and variation in cardiovascular risk factors, medical care, and disease by race, gender, location, and date. To date, the ARIC project has published over 800 articles in peer-reviewed journals. ARIC includes two parts: the Cohort Component and Community Surveillance Component.

The ARIC Cohort Component began in 1987, and each ARIC field center randomly selected and recruited a cohort sample of approximately 4,000 individuals aged 45–64 from a defined population in their community, to receive extensive examinations, including medical, social, and demographic data. Follow-up also occurs semi-annually, by telephone, to maintain contact and to assess health status of the cohort.

In the Community Surveillance Component, the four communities are investigated to determine the long term trends in hospitalized myocardial infarction (MI) and coronary heart disease (CHD) deaths in approximately 470,000 men and women aged 35–84 years.

*Cardiovascular Health Study (CHS).* The CHS [15] is an NHLBI-funded observational study of risk factors for cardiovascular disease in adults 65 years or older. Starting in 1989, and continuing through 1999, participants underwent annual extensive clinical examinations. Measurements included traditional risk factors such as blood pressure and lipids as well as measures of subclinical disease, including echocardiography of the heart, carotid ultrasound, and cranial magnetic-resonance imaging (MRI). At six month intervals between clinic visits, and once clinic visits ended, participants were contacted by phone to ascertain hospitalizations and health status. The main outcomes are coronary heart disease (CHD), angina, heart failure (HF), stroke, transient ischemic attack (TIA), claudication, and mortality. Participants continue to be followed for these events. CHS participants who were free of cardiovascular disease at the start of the study, and who consented to genetic testing, were included in these analyses.

*Erasmus Rucphen Family (ERF) Study.* The ERF study has been described in detail previously [16]. A total of approximately 3,000 participants descend from 22 couples who lived in the Rucphen region in The Netherlands in the 19^th^ century. The 2,755 individuals with genotype data and lipid measurements were included in the current analysis.

*Framingham Heart Study (FHS).* The Framingham Heart Study (FHS), funded by the National Heart Lung and Blood Institute, is an observational population-based cohort study composed of three generations of Framingham (MA) residents predominately of European descent. The Original cohort (*N* = 5,209) was enrolled in 1948. The children and spouses of the Original cohort comprise the Offspring cohort (*N* = 5,124), which was enrolled in 1971–1975 [17]. The Third Generation (*N* = 4,095) consists mostly of the children of the Offspring cohort, and was enrolled in 2002 to 2005 [18]. All participants were examined every 4–8 years. DNA for surviving participants was collected in the late 1990s and early 2000s (1995–2005). Cholesterol and genetic data from 3,464 Offspring subjects and 3,569 Third Generation subjects contribute to this paper.

*Northern Finland Brith Cohort 1966 (NFBC-66).* The Northern Finland Birth Cohort 1966 (NFBC-66) study [19] is a longitudinal one-year birth cohort study designed to study the risk factors of perinatal deaths and low birth weight. Mothers living in the two northern-most provinces of Finland were invited to participate if they had expected delivery dates during 1966. Individuals still living in Helsinki area or Northern Finland were asked at age 31 to participate in a detailed examination (*N* = 5,923). Extensive data on intermediate phenotypes related to obesity and behavioral traits have also been collected.

### Genotyping and imputation

All cohorts were genotyped using commercially available Affymetrix or Illumina genotyping arrays, or custom Perlegen arrays. Quality control was performed independently for each study. To facilitate meta-analysis, each replication cohort performed genotype imputation using BIMBAM, IMPUTE, or MaCH with reference to HapMap or the 1000 Genomes project data.

The first two cohorts of the Rotterdam Study were genotyped using the Illumina 550 K chip, the third cohort was genotyped using the Illumina 610 K and 660 K chip. The following exclusions were applied to identify a final set of SNPs that was used in this study: MAF<0.05, SNP callrate <0.95 and/or HWE *p*-value<1 · 10^−7^. The QC was done per cohort.

In ARIC, genotyping was performed with the Affymetrix 6.0 chip. After genotyping, the following quality control tresholds were applied: (1) comparison of genotype calls to sample replicates, with exclusion of samples with greater than 1% mismatch, (2) exclusion of samples with greater than 5% missing genotypes, (3) exclusion of samples with a mismatch between reported sex and that determined by genotyping, (4) exclusion of SNPs with greater than 10% missing genotypes across samples, (5) exclusion of SNPs monomorphic in both races and (6) exclusion of SNPs (MAF>0.05) with HWE *p*-values of less than 1 · 10^−6^. Prior to imputations, principal component analysis was performed to exclude outliers. Imputation to HapMap release 23a was performed using MaCH v.1.0. After imputation SNPs with an imputation quality less than 0.90 were excluded. 26.8% of the SNPs in the replication were genotyped, the rest was imputed.

In AGES only imputed SNPs were used for the replication. The genotypes originated on Illumina Hu370CNV. For imputation, only the SNPs were included which were completed in 97% of individuals and had a MAF above 1%. Imputation was performed by MaCH against HapMap Release 22. Quality of the imputations was evaluated by the MaCH R^2^ metric.

In CHS, genotyping was performed at the General Clinical Research Center’s Phenotyping/Genotyping Laboratory at Cedars-Sinai using the Illumina 370CNV BeadChip system. Genotypes were called using the Illumina BeadStudio software. The following exclusions were applied to identify a final set of 306655 autosomal SNPs that were used for imputation: call rate <97%, HWE *p*<1 · 10^−5^, >2 duplicate errors or Mendelian inconsistencies (for reference CEPH trios), heterozygote frequency = 0 and SNP not found in HapMap. Imputation to HapMap release 22 (build 36) was performed using BimBam v.0.99. Most of the replication SNPs were genotyped (58.4%), the remaining were imputed.

In ERF genotyping was done on various Illumina and Affymetrix chips. QC was done for each chip separately. On average, the following QC criteria were applied: callrate >0.98, per individual callrate >0.96, HWE *p*-value>5 · 10^−8^ and MAF>0.005. IBS checks, sex chromosome checks and ethnicity checks were also performed. The imputation to Hapmap 2 release 22 was performed with MaCH and minimac. All SNPs in the replication were imputed.

In FHS genotyping was done on Affymetrix 250 K Nsp and 250 K Sty mapping arrays and the Affymetrix 50 K supplemental gene-focused array. The following QC criteria were applied before imputations: *p*_HWE_<1 · 10^−6^, callrate >0.97, mishap test of non-random missingness *p*<1 · 10^−9^, <100 Mendelian errors. The genotyped SNPs were imputed against HapMap (release 22, build 36, CEU population) with MaCH (version 1.0.15). All SNPs in the replication were imputed.

In NFBC-66 genotyping was done on Illumina 370 K whole-genome SNP array. The following QC criteria were observed: SNP clustering probability of genotypes >95%, sample call rate >95%, SNP call rate >95%, MAF>1% and HWE *p*-value>1 · 10^−6^. Heterozygosity, gender check and relatedness checks were performed and any discrepancies were removed. 10 individuals with cryptic relatedness were also excluded from the analysis. To identify a final set of SNPs for imputations, a SNP call rate filter of >99% was applied to all SNPs with MAF<5%. The imputation to 1000 Genomes Phase I integrated variant set (Mar 2012) was performed using IMPUTE v2.2.2. After imputation only those variants with info score >0.9 were analysed. 58.6% of the SNPs in the replication were genotyped, the rest was imputed.

### Study samples and phenotypes

A summary of the details of the nine studies participating in this analysis can be found in Table 1. In all studies, the subjects were fasting when the HDL levels were measured. The HDL measurements were adjusted for sex and age, except for NFBC-66 in which only was adjusted for sex since all individuals are from the same age. In ERF mmscore (GenABEL version 1.7.0 [20]) was used to account for family relationships. In ARIC, the HDL levels were also adjusted for the three ARIC field center with two 0,1 indicator variables. In CHS the HDL was adjusted for study clinic site as well and in NFBC-66 HDL was also adjusted for 10 PC components. In FHS the HDL levels were also adjusted for related individuals with the lmekin function within the coxme package in R (http://cran.r-project.org/web/packages/coxme/) and adjusted for PCs. In the discovery and filtering stage, the HDL levels after adjustment for sex and age were normalised around zero as this is a requirement of GLIDE. To compare the β_int_ in the discovery and filtering stage with the ß_int_ in the replication stage, we also calculated the β_int_ in the Rotterdam Study cohorts without scaling around zero for the most promising interaction terms.

### GWIS with GLIDE in RS-I

To systematically search for the epistatic interactions associated with HDL levels in RS-I we used GLIDE [12]. GLIDE makes use of the computational power of consumer-grade graphics cards to detect interactions between SNPs via linear regression. To reduce computation time, we chose to run GLIDE on genotyped SNPs only. In order to run GLIDE, the genotype data of RS-I was stored per chromosome as a text file with one row per SNP and one column per individual. Individuals using lipid-lowering medication were excluded. The file does not contain column headers or row names and the SNPs need to be coded 0 (homozygous for the major allele), 1 (heterozygous) or 2 (homozygous for the minor allele). We only used SNPs with a MAF (Minor Allele Frequency) >0.05 within the samples of RS-I, RS-II and RS-III which were used in this study, since the sample size is not large enough to investigate low-frequency variants.

The names of the SNPs are stored in a separate one-column text file in the same order as the SNPs in the file with the genotype data. The values of the scaled residuals are stored in a separate text file in the same order as the individuals in the file with the genotype data. GLIDE requires the phenotype to be normalised around zero. GLIDE uses the files with the genotypes and the file with the scaled residuals to perform linear regression for all possible unique SNP×SNP combinations. In order to fit the data into the GPU’s memory, GLIDE splits up the genotypes in subsets of SNPs. In this study we chose to split up in subsets of 1000 SNPs. GLIDE outputs a *t*-score for each interaction term and a threshold can be set to only output interactions with a *t*-score above this threshold.

The output of GLIDE does not contain the SNP names, but the number of the chunk and the number of the SNP within a given chunk. With help of the previously created SNP files, we assigned SNP names to the interaction terms output by GLIDE. Since GLIDE handles the data in chunks, interaction terms occur multiple times in the output of GLIDE, consequently, the results had to be filtered on unique interaction terms.

### Filtering of interaction terms by meta-analysis of RS-I, RS-II and RS-III

To reduce the number of false positive interaction terms, we filtered the interaction terms with an absolute value of the *t*-score >5 (*p*-value<6.06 · 10^−7^) by a meta-analysis of RS-I, RS-II and RS-III. For these interactions, we used linear regression to determine the ßs, standard errors and *p*-values in RS-I, RS-II and RS-III. The HDL levels after adjustment for sex and age were normalised around zero in all three cohorts. The βs and standard errors of all three cohorts of the Rotterdam Study were subsequently meta-analyzed to filter out only those with a *p*-value less than 1 · 10^−8^.

### Replication of SNP×SNP interactions

The interaction terms which had a *p*-value less than 1 · 10^−8^ after meta-analysis of the three Rotterdam Study cohorts, were replicated in 6 cohorts: AGES, ARIC, CHS, ERF, FHS and NFBC-66. Only individuals that do not use lipid-lowering medication were included, except for AGES. The linear regression model for replication was
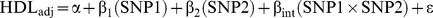
where HDL_adj_ are the HDL levels adjusted for sex and age. We meta-analysed the β_int_ from all 6 replication cohorts.

To see if the filtered interaction terms effect the probability of using lipid-lowering medication, we performed a case-control study in the three Rotterdam Study cohorts. Those individuals that have HDL levels available and use lipid-lowering medication were defined as cases and the individuals in the discovery or filtering stage were defined as controls. The logistic regression model for replication was
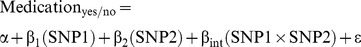



We performed the analysis in the three cohorts separately, and also in the three cohorts combined, in which we included the cohort number as an additional covariate.

### Power calculations

To estimate the effect we could have detected with the current sample size, a certain type I error and various type II erros, we used G*Power [21], [22] (version 3.1.9.2).

## Supporting Information

Table S1 The 181 interaction terms with a *p-*value<1 · 10^−8^ after meta-analysis in RS-I, RS-II and RS-III.
(XLS)
